# Overexpression of NPTX2 Promotes Malignant Phenotype of Epithelial Ovarian Carcinoma *via* IL6-JAK2/STAT3 Signaling Pathway Under Hypoxia

**DOI:** 10.3389/fonc.2021.643986

**Published:** 2021-03-09

**Authors:** Xiaotian Han, Yechen Lu, Xiaoqi Li, Lingfang Xia, Hao Wen, Zheng Feng, Xingzhu Ju, Xiaojun Chen, Xiaohua Wu

**Affiliations:** ^1^Department of Gynecologic Oncology, Fudan University Shanghai Cancer Center, Shanghai, China; ^2^Department of Oncology, Shanghai Medical College, Fudan University, Shanghai, China; ^3^Wound Repair Center, Ruijin Hospital, Shanghai Jiaotong University, Shanghai, China

**Keywords:** NPTX2, epithelial ovarian carcinoma (EOC), IL6-JAK2/STAT3 signaling pathway, malignant phenotype, hypoxia

## Abstract

**Background:**

Epithelial ovarian cancer (EOC) is the main subtype of ovarian cancer and shows an aggressive phenotype and poor prognosis. Neuronal pentraxin II (NPTX2) is a member of the neuronal pentraxin family and plays a contradictory role in different tumors. However, there has been no report about the possible role and effect of NPTX2 in EOC.

**Methods:**

Bioinformatics analysis, qPCR, western blotting and immunohistochemistry were used to detect the expression of NPTX2 in EOC. Lentivirus-based transfection for NPTX2 overexpression or knockdown was performed on the EOC cell lines A2780, HEY, SKOV3 and OVCAR-3. The effect of NPTX2 on the malignant phenotype of EOC was examined through methods of MTS assay, Edu assay, transwell assay, western blotting analysis, qPCR analysis, luciferase reporter assay and xenograft experiment.

**Results:**

EOC tissues showed higher NPTX2 expression than the normal tissues with poor prognosis. NPTX2 overexpression can promote the proliferation, invasion, migration and tumorigenesis of EOC *via* IL6-JAK2/STAT3 signaling pathway. Moreover, hypoxia-inducible factor-1(HIF-1) can promote the transcription and expression of NPTX2 under the hypoxic environment. NPTX2 knockdown abolished the hypoxia-induced malignant phenotypes in ECO.

**Conclusions:**

The above results suggest that NPTX2 may play a novel role in ovarian cancer’s malignant phenotype and act as a promising treatment target for EOC molecular therapy.

## Introduction

Ovarian cancer is one of the most common malignant tumors in females with the worst prognosis, accounting for 3.4% of all the female malignant tumors and 4.4% of female cancer deaths (1). Mostly, epithelial ovarian cancer (EOC) is the main subtype of ovarian cancer and shows a more aggressive phenotype than nonepithelial cancers (2). Although surgery combined with chemotherapy has achieved a certain effect, the patients’ long-term prognosis is not ideal. It has been reported that the 5-year survival rate is only 29% in the advanced stage of EOC patients (3). At present, molecular targeted therapy is regarded as one of the most promising therapies for EOC treatment (4). Identifying genes associated with tumorigenesis, proliferation, invasion and migration of EOC has significant implications for EOC clinical treatment and basic research (5).

Neuronal pentraxin II (NPTX2), also known as neuronal activity-regulated pentraxin, is a member of the neuronal pentraxin family (6). NPTX2 was initially expressed in the central nervous system (CNS) and played a role in promoting excitatory synapse formation and synaptic remodeling (6, 7). NPTX2 is highly upregulated and participates in developing several CNS diseases, such as Parkinson’s disease (8). Moreover, several studies have also reported that NPTX2 is involved in the occurrence and development of malignant tumors, while it plays a contradictory role in different tumors. For example, NPTX2 promotes colorectal cancer growth and liver metastasis by activating the canonical Wnt/β-catenin pathway *via* FZD6 (9). NPTX2 overexpression promotes the cell proliferation, migration and invasion of renal cell carcinoma (10). However, NPTX2 was down-regulated and correlated with the poor prognosis of pancreatic cancer and glioma due to its promoter hypermethylation (11, 12). Although NPTX2 was found to be expressed in female reproductive organs, such as the ovary, cervix and uterine, according to the human protein atlas (13), there has been no report about the possible role and effect of NPTX2 in EOC.

In the present study, we first found abnormally higher NPTX2 expression in EOC using bioinformatics analyses based on the TCGA database and four GEO datasets (GSE10971, GSE18520, GSE105437, and GSE26712), followed with clinical specimens’ validation. Higher NPTX2 expression was correlated with the poor prognosis of EOC. Therefore, NPTX2 maybe a novel oncogene in the malignant of EOC and the aim of our study was to investigate the role and demonstrate the potential mechanism of NPTX2 in EOC.

## Materials and Methods

### Patients and Samples

In the present study, patient samples and adjacent non-tumor tissues were taken from 45 patients diagnosed with EOC *via* pathology and underwent surgical excision without prior anticancer treatments at Fudan University Shanghai Cancer Center between February 2018 to May 2019. The detailed clinical features of these patients were listed in **Supplementary Table 1**. This study was approved by the institutional review board of the Fudan University Shanghai Cancer Center.

### Cell Treatment

The human EOC cell lines OVCA420, A2780, and OVCAR3 were purchased from the Chinese Academy of Sciences cell bank (Shanghai, China). The wild-type human ovarian epithelial cell line HS832.Tc and human EOC cell lines SKOV3, HEY were purchased from American Type Culture Collection (ATCC, Manassas, VA, USA). All cells were maintained in Dulbecco’s modified Eagle’s medium (DMEM, HyClone, Logan, UT, USA) supplemented with 10% fetal bovine serum (FBS, Gibco, Carlsbad, CA, USA) and 1% penicillin/streptomycin (Gibco) at 37°C with 5% CO_2_. To induce hypoxia, cells were cultured in a hypoxia chamber with 94% N_2_, 5% CO_2_, and 1% O_2_ at 37°C. IL-6-neutralizing antibody (R&D Systems, Minneapolis, MN, USA) was used at a concentration of 0.5 μg/ml (14). All cells analyzed were cultured with less than 20 generations.

### Lentiviral Vector Construction and Transfection

Lentivirus-based vector transfection for NPTX2 overexpression and RNAi-mediated NPTX2 knockdown was performed with technical support from Gene-Chem (Shanghai, PR China). Two siRNA sequences were designed for NPTX2 knockdown: forward 5′- ACUUAAAGGCGCUAUUGCCUC-3′, reverse 5′- GGCAAUAGCGCCUUUAAGUCA -3′ and forward 5′- UGACUUAAAGGCGCUAUUGCC -3′, reverse 5′- CAAUAGCGCCUUUAAGUCACC -3′. After transfection, all cells were treated with puromycin (Sigma, Santa Clara, CA, USA) at a concentration of 10μg/ml for 30 days. Both qPCR and western blotting were used to verify the effectiveness of NPTX2 overexpression or knockdown.

### qRT-PCR (Real-Time Quantitative Reverse Transcription PCR)

The total RNA of EOC tissues and cell lines were isolated *via* the Mini-BEST Universal RNA Extraction kit (TaKaRa, Kyoto, Japan) according to the manufacturer’s instructions, followed with first-strand cDNA synthesis *via* Prime-Script RT Master Mix (TaKaRa). The qPCR detection was performed using the SYBR Green Master Mix (TaKaRa) *via* PCR LightCycler480 (Roche Diagnostics Ltd., Basel, Switzerland). Each sample was run four times and β-actin was used as ;the internal control. The sequences of the qPCR primer pairs were as follows: NPTX2, forward 5′ - ACGGGCAAGGACACTATGG-3′ and reverse 5′ - ATTGGACACGTTTGCTCTGAG-3′; IL6, forward 5′ - ACTCACCTCTTCAGAACGAATTG-3′ and reverse 5′ - CCATCTTTGGAAGGTTCAGGTTG-3′; β-actin, forward 5′ -CATGTAC GTTGCTATCCAGGC-3′ and reverse 5′ -CTCCTTAAT GTCACGCACGAT-3′.

### Western Blotting

The total protein of EOC tissues and cell lines was isolated *via* a cell protein extraction kit (KeyGen Biotechnology, Nanjing, PR China) according to the manufacturer’s instructions. The BCA kit (Beyotime Biotechnology, Beijing, PR China) was used to determine the concentration. An equivalent amount of protein from each sample was separated by 4 to 20% SDS-PAGE (Genscript, Nanjing, China), transferred to a nitrocellulose membrane and blocked with 2% bovine serum albumin (KeyGen Biotechnology). The membrane was then incubated with primary antibodies against NPTX2, IL6, p-JAK2, JAK2, p-STAT3, STAT3 and β-actin (Abcam Technology, Cambridge, UK) at 4°C overnight, followed with TBST washing and incubated with secondary antibody. The ECL kit (Beyotime) was used to detect the bands on each membrane, and IMAGE J software (National Institutes of Health, Bethesda, MD, USA) was used for quantification.

### Cell Viability Assay

According to the manufacturer’s instructions, the CellTiter 96^®^ AQueous Non-Radioactive cell proliferation assay kit (Promega, Madison, WI, USA) was used for cell viability detection. Briefly, EOC cells were cultured in 96-well plates at a density of 3 × 10^3^ cells/well for 24, 48, 72, 96, and 120 h. Then 20μl of MTS was added into each well, followed with 1 h incubation at 37°C. An ultraviolet spectrophotometer (Thermo Fisher Scientific, Waltham, MA, USA) was used to detect the absorbance at 495 nm.

### Edu Assay

The 5-ethynyl-20-deoxyuridine (Edu) incorporation assay (Beyotime) was performed according to the manufacturer’s instructions. EOC cells were seeded into 24-well plates at a density of 1 × 105 cells per well for 20 h, followed with 50 μMEdU treatment for 2 h at 37°C. The cells were then fixed with 4% paraformaldehyde, permeabilized with 0.3% Triton X-100, incubated with 100 μl Click Additive Solution for 30 min, and stained with 100 μl DAPI. The images were taken with a laser scanning confocal microscope (Olympus), and the percentage of Edu-positive cells was calculated.

### Transwell Assay

For the transwell assay, the 8 μm pore size polycarbonate membrane of the upper transwell chamber (Costar, Corning, NY, USA) was covered with 80 μL of 50 ng/μL Matrigel solution (BD, Franklin Lakes, NJ, USA) for 1 h incubation at 37°C. Then the EOC cells were resuspended in serum-free DMEM at a density of 2 × 10^5^ cells/mL, and then 100 μL of cell suspension was seeded into the upper chamber, and 600 μL DMEM with 20% FBS was added to the lower chamber. After incubation at 37°C for 20 h, the cells were fixed with 4% paraformaldehyde (Beyotime) for 10 min and stained with 1% Crystal Violet solution (Beyotime) for 20 min at room temperature. Finally, the cell numbers were counted by calculating the average of five random fields using an inverted microscope (Olympus). For the migration assay, the upper transwell chamber was not treated with Matrigel solution, and the other steps were the same as the transwell assay.

### Immunohistochemistry (IHC)

Immunohistochemistry was performed using an immunohistochemistry kit (Maixin Biotechnology, Fuzhou, Fujian, PR China) according to the manufacturer’s instructions. Briefly, tissue samples were paraffin-embedded, cut into 4-μm sections, and incubated with primary antibodies against NPTX2 and Ki-67 (Abcam) at 4°C overnight. After DAB staining, the sections were imaged with an optical microscope (Olympus), and staining intensity was assessed according to the German immunohistochemical scoring system (15).

### Luciferase Activity Analysis

Luciferase reporter assays were performed using the Dual-Luciferase Reporter Assay System (Promega) according to the manufacturer’s instructions. Briefly, NPTX2 reporter plasmids were constructed by Gene-Chem (Shanghai, PR China). EOC cells were seeded into 96-well plates at a density of 3 × 10^3^ cells per well and transfected with different plasmids. The cells were lysed after 48 h, and luciferase activity was measured using an ultraviolet spectrophotometer (Thermo Fisher Scientific). Each experiment was independently repeated three times.

### Chromatin Immunoprecipitation (ChIP) Assays

ChIP assays were performed using the EZ-ChIP ™ Immunoprecipitation Kit (Millipore, Billerica, MA, USA) according to the manufacturer’s instructions. The chromatin complexes were immuno-precipitated with anti-HIF-1 antibody (Abcam). Then the ChIP Assay Kit (Beyotime) was used to purify DNA samples, and the purified DNA was analyzed by qPCR. The primer pairs used to amplify the HIF-1 binding site in the NPTX2 promoter was: f: 5′- GTGACACTGCGGGCCCT-3′ and r: 5′- CGTCCGGCGCCTGGTCCT-3′.

### Xenograft Experiments

Five-week-old female BALB/c nude mice were purchased from Shanghai Laboratory Animal Center and bred in the Laboratory Animal Center of Fudan University Shanghai Cancer Center under specific-pathogen-free conditions. Mice were maintained under a 12 h light/12 h dark cycle with free access to water and standard mouse diet. EOC cell suspensions (5 × 10 ^5^ cells/100 μL of DMEM) were mixed with 100 μL of Matrigel solution (BD) and injected into the back flanks of the nude mouse. A2780 with lower NPTX2 expression was used for overexpression, while OVCAR-3 with higher NPTX2 expression was used for knockdown. The tumor size was measured using a Vernier caliper once a week, and the tumor volume was calculated using the following formula: V = (D × d)/2 mm, where D is the longest diameter and d is the shortest diameter of the tumor. The mice were sacrificed 5 weeks after implantation, and the tumors were weighed and photographed.

### Bioinformatic Analyses

The data on mRNA expression and clinical samples from ovarian carcinoma patients were obtained from The Cancer Genome Atlas (TCGA, http://cancergenome.nih.gov) and Gene Expression Omnibus (GEO, GSE10971, GSE18520, GSE105437 and GSE26712). Gene-set enrichment analysis (GSEA, http://www.broadinstitute.org/gsea/index.jsp) was used to detect any possible signal pathway sets of genes showing statistically significant differences between higher and lower NPTX2 expression groups.

### Statistical Analysis

Statistical analysis was performed using SPSS 23.0 software (IBM, Armonk, N.Y, USA). All experiments were repeated at least three times, and the results are presented as the mean ± SD. The chi-square test, two-tailed Student’s t-test and one-way analysis of variance were used to compare the statistical significance among different groups. Differences in survival rates were analyzed using the log-rank test and Kaplan-Meier analysis. All statistical tests were two-sided, and statistical significance was defined as a p-value < 0.05.

## Results

### NPTX2 Is Upregulated in Epithelial Ovarian Carcinoma and Correlated With the Progression and Poor Prognosis

**Figure 1:**
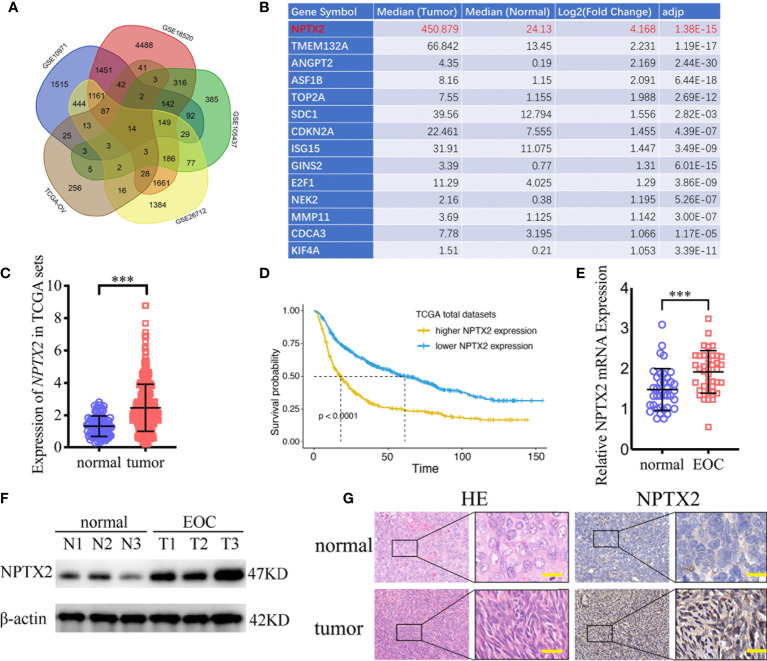
NPTX2 is upregulated in ovarian carcinoma and correlated with the progression and poor prognosis. **(A, B)** Fourteen upregulated genes with P<0.05 were found in the ovarian carcinoma based on TCGA and four GEO databases (GSE10971, GSE18520, GSE105437, and GSE26712). **(C)** The expression of NPTX2 in ovarian carcinoma tissues and normal ovarian tissues according to the TCGA database. **(D)** The prognostic significance of NPTX2 in ovarian carcinoma was confirmed in the TCGA database. **(E, F)** NPTX2 is expressed at higher levels in EOC tissues than the normal tissues as measured by qPCR **(E)** and western blotting **(F)**. **(G)** Representative immunohistochemical staining for NPTX2 in EOC tissues and the normal tissues. All data are shown as the mean ± SD (three independent experiments). ***P < 0.001.

**Figure 2:**
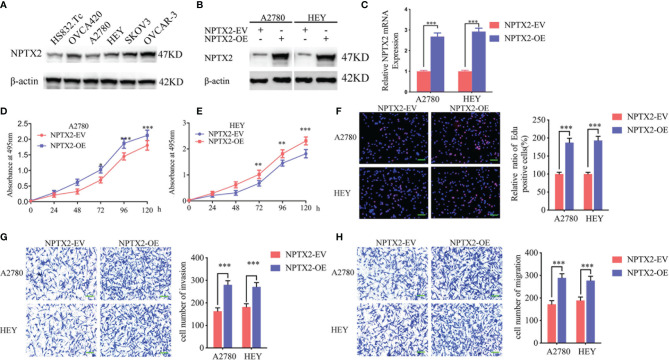
NPTX2 overexpression promoted ovarian carcinoma proliferation *in vitro*. **(A)** Western blotting showing the expression of NPTX2 in EOC cell lines and wild-type human ovarian epithelial cell line.**(B, C)** Lentiviral-based transfection and the effects on NPTX2 overexpression were validated by western blotting **(B)** and qPCR **(C)**. **(D, E)** The cell viability of A2780 **(D)** and HEY **(E)** increased after NPTX2 overexpression as measured by the MTS assay. **(F)** The proliferation of the A2780 and HEY increased after NPTX2 overexpression as measured by the EDU incorporation assay, scale bar=100 μm. **(G, H)** Representative transwell assay **(G)** and migration assay **(H)** showing the increase in invasion and migration of A2780 and HEY after NPTX2 overexpression. Scale bar=100 μm. EV, empty vector; OE, overexpression, +: treated; −: untreated. All data are shown as the mean ± SD (three independent experiments). ***P < 0.001.

To find the oncogenes that may participate in the carcinogenesis and development of ovarian carcinoma, we first searched TCGA and four GEO databases, including GSE10971, GSE18520, GSE105437, and GSE26712, and found that 14 genes were upregulated in these five databases (**Figure 1A**). NPTX2 is the most significantly upregulated gene among these genes, while its biological role in ovarian cancer has not been reported (**Figures 1B, C**). In addition, we performed Kaplan-Meier survival analysis for the prognostic significance of NPTX2 expression in ovarian carcinoma patients, and the survival time of patients with high NPTX2 expression was significantly lower in the TCGA dataset than in patients with low expression (**Figure 1D**). We then detected clinical specimens from ovarian carcinoma patients and found the similar NPTX2 expression as that in TCGA and GEO databases. All qPCR (**Figure 1E**), western blotting (**Figure 1F**) and immunohistochemistry (**Figure 1G**) showed NPTX2 expressed higher in epithelial ovarian carcinoma than the normal tissues. Moreover, we also detected NPTX2 expression in EOC cell lines OVCA420, A2780, SKOV3, HEY, and OVCAR-3 and wild-type human ovarian epithelial cell line HS832.Tc. All EOC cell lines showed higher NPTX2 expression than the benign cell line HS832.Tc (**Figure 2A**). The above results demonstrated that NPTX2 was upregulated in ovarian carcinoma and correlated with the progression and poor prognosis.

### NPTX2 Overexpression Promoted the Malignant Phenotype of Epithelial Ovarian Carcinoma *In Vitro*

To investigate the role of NPTX2 in EOC, we designed lentiviral-encapsulated NPTX2 overexpression plasmids. According to the expression of NPTX2 in EOC cell lines shown in **Figure 2A**, A2780 and HEY with the lowest expression were treated for NPTX2 overexpression. Both western blotting and qPCR confirmed the overexpression of NPTX2 (**Figures 2B, C**). Then MTS assays showed the absorbance values were significantly higher than controls (**Figures 2D, E**), confirming that NPTX2 overexpression promoted EOC cell lines’ cell viability. Edu assays showed the Edu positive rates were significantly higher than controls (**Figure 2F**), confirming that NPTX2 overexpression promoted EOC cell lines’ proliferation. Both transwell and migration assays showed that NPTX2 overexpression markedly promoted tumor cell invasion and migration compared with controls (**Figures 2G, H**). The above results confirm that NPTX2 overexpression promoted the malignant phenotype of epithelial ovarian carcinoma *in vitro*.

### NPTX2 Knockdown Inhibited Malignant Phenotype of Epithelial Ovarian Carcinoma *In Vitro*

**Figure 3:**
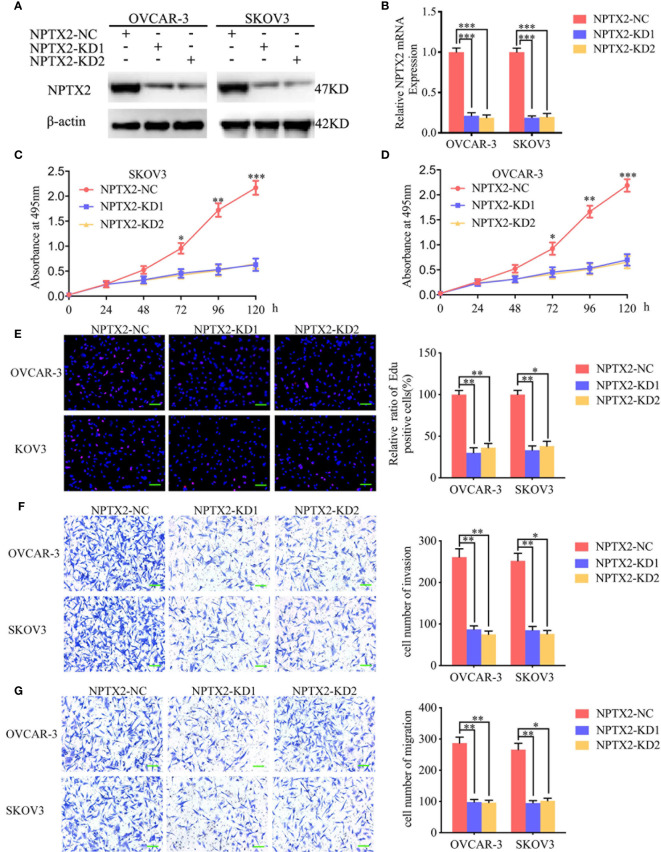
NPTX2 knockdown inhibited ovarian carcinoma proliferation *in vitro*. **(A, B)** Lentiviral-based transfection and the effects on NPTX2 knockdown were validated by western blotting **(A)** and qPCR **(B)**. **(C, D)** The cell viability of SKOV3 **(C)** and OVCAR-3 **(D)** decreased after NPTX2 knockdown as measured by the MTS assay. **(E)** The proliferation of the SKOV3 and OVCAR-3 decreased after NPTX2 knockdown as measured by the EDU incorporation assay, scale bar=100 μm. **(F, G)** Representative transwell assay **(F)** and migration assay **(G)** showing the inhibition of the invasion and migration of SKOV3 and OVCAR-3 after NPTX2 knockdown. Scale bar=100 μm. NC, negative control; KD, knockdown, +: treated; −: untreated. All data are shown as the mean ± SD (three independent experiments). *P < 0.05; **P < 0.01; ***P < 0.001.

To further demonstrate the role of NPTX2 in the proliferation and metastasis of EOC, we designed two siRNA sequences and transfected OVCAR-3 and SKOV3 to knockdown NPTX2. Western blotting and qPCR were used to confirm the efficiency of NPTX2 knockdown (**Figures 3A, B**). First, we used the MTS assay to detect tumor cells’ proliferation activity after NPTX2 knockdown, and the absorbance values were significantly lower than those of the control group (**Figures 3C, D**), confirming that NPTX2 knockdown inhibits the cell viability of EOC cell lines. Edu assays showed the Edu positive rates were significantly lower than controls (**Figure 3E**), confirming that NPTX2 knockdown inhibits EOC cell lines’ proliferation. Subsequently, we examined the effect of NPTX2 knockdown on tumor cell metastasis by both transwell and migration assays. As shown in **Figures 3F, G**, NPTX2 knockdown markedly inhibited tumor cell invasion and migration compared with controls. Taken together, these results clearly demonstrated that NPTX2 knockdown inhibited the malignant phenotype of epithelial ovarian carcinoma *in vitro*.

### NPTX2 Regulates IL6-JAK2/STAT3 Signaling Pathway in Epithelial Ovarian Carcinoma

**Figure 4:**
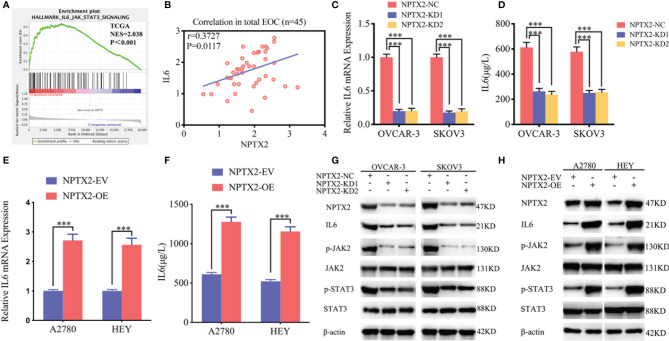
NPTX2 regulates IL6-JAK2/STAT3 signaling pathway in ovarian carcinoma. **(A)** Gene set enrichment analysis (GSEA) indicates that high expression of NPTX2 is associated with the IL6-JAK2/STAT3 signaling pathway in the ovarian carcinoma TCGA database. **(B)** qPCR indicates a positive correlation between NPTX2 and IL6 expression in EOC clinical patients. **(C, D)** The expression and secretion of IL6 after NPTX2 knockdown in SKOV3 and OVCAR-3 was measured by qPCR **(B)** and ELISA **(C)**. **(E, F)** The expression and secretion of IL6 after NPTX2 overexpression in A2780 and HEY was measured by qPCR **(D)** and ELISA **(E)**. **(G, H)** The major molecules in the IL6-JAK2/STAT3 signaling pathway after NPTX2 knockdown **(F)** or overexpression **(G)** was measured by western blotting. EV, empty vector; OE, overexpression; NC, negative control; KD, knockdown; +: treated; −: untreated. All data are shown as the mean ± SD (three independent experiments). ***P < 0.001.

To further explore the possible signaling pathways in which NPTX2 regulates the proliferation, invasion and migration of EOC, we performed GSEA on the TCGA dataset. The results showed a significant IL6-JAK2/STAT3 signaling pathway enrichment in the higher NPTX2 expression group (**Figure 4A**). Therefore, we first detected the expression of NPTX2 and IL6 in EOC clinical patients *via* qPCR and the results demonstrated there was a positive correlationship (**Figure 4B**). Then we examined the changes of IL6 after NPTX2 regulation. Therefore, we first examined changes of IL6 after NPTX2 regulation. All the ELISA, qPCR, and western blotting results showed the expression and secretion of IL6 were obviously down-regulated after NPTX2 knockdown in OVCAR-3 and SKOV3 (**Figures 4C, D, G**), while the opposite results were got after NPTX2 overexpression in A2780 and HEY (**Figures 4E, F, H**). Then the downstream molecules of the IL6-JAK2/STAT3 signaling pathway were detected by western blotting. As shown in **Figure 4F, G**, the expression levels of the p-JAK2 and p-STAT3 were significantly downregulated after NPTX2 knockdown in OVCAR-3 and SKOV3 (**Figure 4G**), while the opposite results were obtained after NPTX2 overexpression in A2780 and HEY (**Figure 4H**). The above evidence proves that NPTX2 can upregulate IL6 expression and activate the JAK2/STAT3 signaling pathway in epithelial ovarian carcinoma.

### NPTX2 Promoted the Malignant Phenotype of Epithelial Ovarian Carcinoma Via IL6-JAK2/STAT3 Signaling Pathway

**Figure 5:**
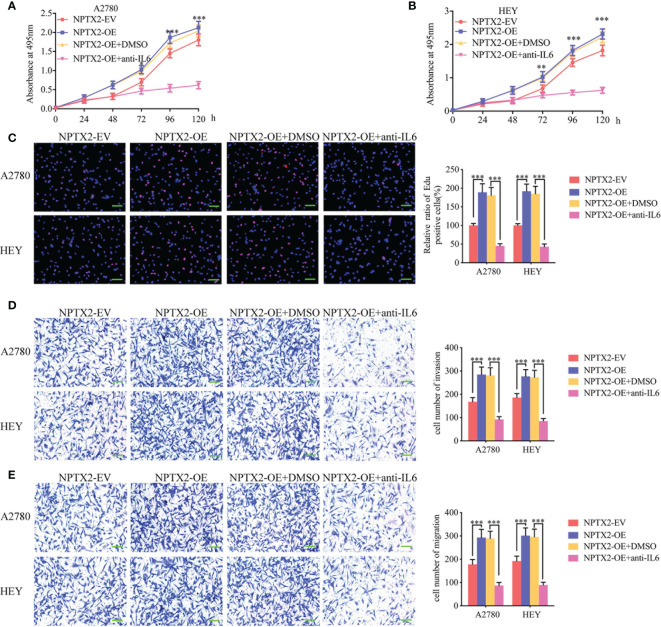
NPTX2 promoted ovarian carcinoma proliferation *via* IL6-JAK2/STAT3 signaling pathway. **(A, B)** MTS assays showing the cell viability of NPTX2 overexpressed A2780 **(A)** and HEY **(B)** were reversed after IL-6-neutralizing antibody treatment. **(C)** EDU incorporation assay showing the proliferation of the NPTX2 overexpressed A2780 and HEY were reversed after IL-6-neutralizing antibody treatment. Scale bar=100 μm. **(D, E)** Representative transwell assay **(D)** and migration assay **(E)** showing the increase in invasion and migration of NPTX2 overexpressed A2780 and HEY were reversed after IL-6-neutralizing antibody treatment. Scale bar=100 μm. EV, empty vector; OE, overexpression; +: treated; −: untreated. All data are shown as the mean ± SD (three independent experiments). *P < 0.05; **P < 0.01; ***P < 0.001.

To furtherly verify whether NPTX2 promotes malignant progression in EOC through the IL6-JAK2/STAT3 signaling pathway, we treated EOC cell lines A2780 and HEY after NPTX2 overexpression with an IL-6-neutralizing antibody. MTS assays showed higher absorbance values caused by NPTX2 overexpression were reversed after IL-6-neutralizing antibody treatment (**Figures 5A, B**). Edu assays showed a higher Edu positive rate during NPTX2 overexpression were reversed after IL-6-neutralizing antibody treatment (**Figure 5C**). Both transwell and migration assays showed that NPTX2 overexpression markedly promoted tumor cell invasion and migration, while these promoting effects were also reversed after IL-6-neutralizing antibody treatment (**Figures 5D, E**). The above results confirm that NPTX2 overexpression promoted the malignant phenotype of epithelial ovarian carcinoma *via* IL6-JAK2/STAT3 signaling pathway.

### HIF-1 Can Directly Induce the Expression of NPTX2 Under Hypoxia

**Figure 6:**
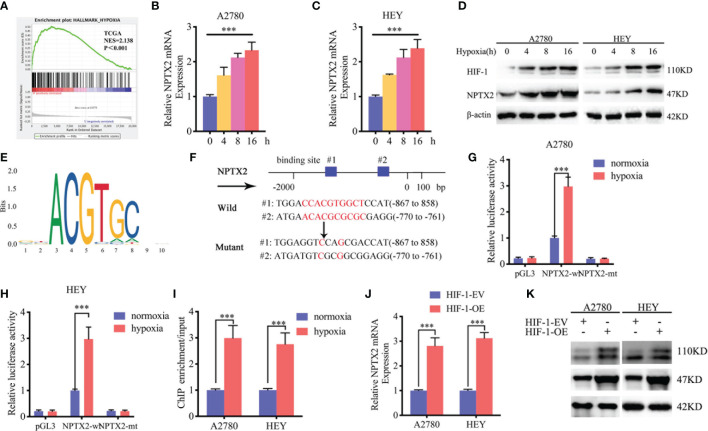
HIF1 can directly induce the expression of NPTX2 under hypoxia. **(A)** Gene set enrichment analysis (GSEA) indicates that high expression of NPTX2 is associated with the hypoxia in the ovarian carcinoma TCGA database. **(B, C)** NPTX2 mRNA expression of A2780 **(B)** and HEY **(C)** gradually increased during prolonged treatment under hypoxia as measured by qPCR. **(D)** NPTX2 protein expression of A2780 and HEY was gradually increased during prolonged treatment under hypoxia as measured by western blotting. **(E)** Sequence motif representing the consensus HIF-1 binding motif (JASPAR database). **(F)** A schematic diagram of the putative HIF-1 binding site in the promoter of NPTX2. **(G, H)** Luciferase reporter assays showed hypoxia can upregulate the luciferase promoter activities of NPTX2 in A2780 **(G)** and HEY **(H)** cells. **(I)** ChIP qPCR showed HIF-1 binding to the promoter of NPTX2 under hypoxia. **(J, K)** qPCR and western blotting showed NPTX2 expression was remarkably upregulated after HIF-1 overexpression. EV, empty vector; OE, overexpression; +: treated; −: untreated. All data are shown as the mean ± SD (three independent experiments). ***P < 0.001.

We furtherly investigated the possible upstream mechanism of NPTX2 overexpression in EOC. GSEA analysis based on the TCGA dataset revealed significant hypoxia enrichment at a higher NPTX2 expression group (**Figure 6A**). Therefore, we investigated whether there is a functional relationship between NPTX2 and hypoxia. The A2780 and HEY cells were treated under hypoxia, and the expression of NPTX2 increased gradually with the extension of the hypoxia treatment time according to both qPCR and western blotting (**Figures 6B–D**). Since hypoxia-inducible factor-1 (HIF-1) is the most important transcription factor under hypoxic conditions (**Figure 6E**), we furtherly discussed whether there is a transcriptional regulation relationship between HIF-1 and NPTX2. We identified the possible binding sites of HIF-1 on the promoter region of NPTX2 by JASPAR analysis (**Figure 6F**), and the luciferase reporter gene assays were performed. The results showed that NPTX2-wt transfected A2780 and HEY had significantly enhanced luciferase activity under hypoxia (**Figures 6G, H**). Moreover, ChIP assays revealed NPTX2 enrichment in A2780 and HEY after anti-HIF-1 treatment under hypoxia (**Figure 6I**). Besides, qPCR and western blotting further confirmed that NPTX2 expression was remarkably upregulated after HIF-1 overexpression(**Figures 6J, K**). In summary, we conclude that HIF-1 directly transcriptionally regulates NPTX2 expression in EOC under hypoxic conditions.

### NPTX2 Knockdown Abolished Hypoxia-Induced Malignant Phenotypes in Epithelial Ovarian Carcinoma

**Figure 7:**
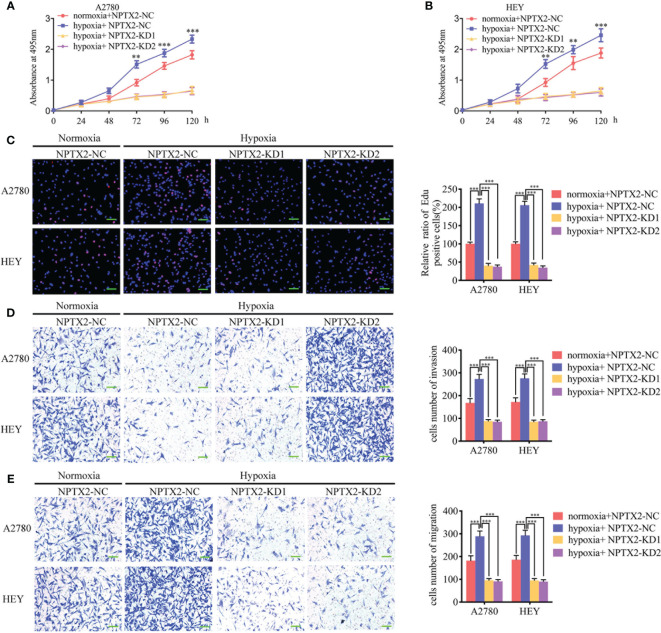
NPTX2 knockdown abolished hypoxia-induced malignant phenotypes in epithelial ovarian carcinoma. **(A, B)** MTS assays showing the cell viability of A2780 **(A)** and HEY **(B)** under hypoxia after NPTX2 knockdown. **(C)** EDU incorporation assay showing the proliferation of A2780 and HEY under hypoxia were reversed after NPTX2 knockdown. Scale bar=100 μm. **(D, E)** Representative transwell assay **(D)** and migration assay **(E)** showing the increase in invasion and migration of A2780 and HEY under hypoxia were reversed after NPTX2 knockdown. Scale bar=100 μm. NC, negative control; KD, knockdown; +: treated; −: untreated. All data are shown as the mean ± SD (three independent experiments). **P < 0.01; ***P < 0.001.

We furtherly detected the function of NPTX2 on epithelial ovarian carcinoma under hypoxia. Both the MTS and EDU assays showed that hypoxic treatment increased cell viability and the rates of EDU-positive cells of A2780 and HEY compared with normoxic GCM treatment (**Figures 7A–C**). However, after NPTX2 knockdown, the increase in proliferation of EOCs induced by hypoxic treatment was abolished (**Figures 7A–C**). Furthermore, both the transwell and migration assays also showed that hypoxic treatment promoted the invasion and migration of EOCs, while NPTX2 knockdown abolished these effects (**Figures 7D, E**). These findings suggested that NPTX2 can mediate the hypoxia-induced epithelial ovarian carcinoma malignant phenotype and NPTX2 can reverse these effects.

### NPTX2 Regulates Epithelial Ovarian Carcinoma Tumorigenesis

**Figure 8:**
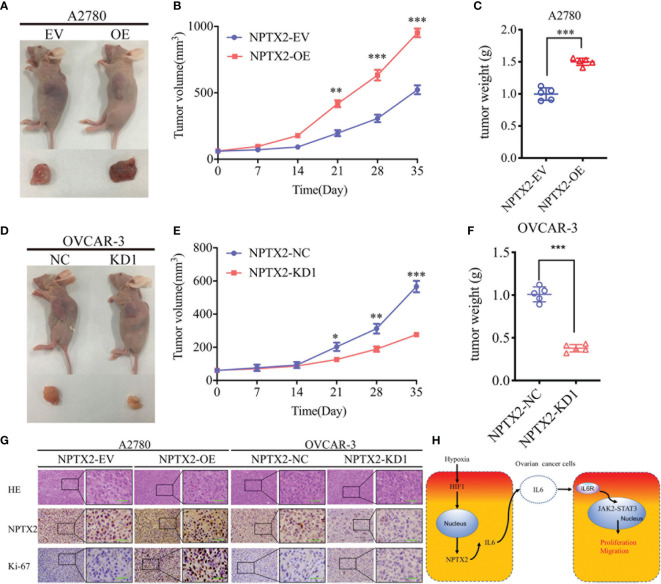
NPTX2 knockdownregulates ovarian carcinoma tumorigenesis. **(A)** Images of subcutaneous xenograft tumors of A2780 cells. **(B, C)** Tumor volume **(B)** and weight **(C)** of A2780 cells was increased after NPTX2 overexpression. **(D)** Images of subcutaneous xenograft tumors of OVCAR-3 cells. **(E, F)** Tumor volume **(E)** and weight **(F)** of OVCAR-3 cells was decreased after NPTX2 knockdown. **(G)** Representative immunohistochemical staining showing the changes in NPTX2 and Ki-67 in NPTX2 overexpression and knockdown subcutaneous xenograft models. Scale bar=50 μm. **(H)** Schematic diagram to illustrate that overexpression of NPTX2 promotes the malignant phenotype in human EOC *via* IL6-JAK2/STAT3 signaling pathway under hypoxia. EV, empty vector; OE, overexpression; NC, negative control; KD, knockdown. *P < 0.05; **P < 0.01; ***P < 0.001.

Finally, to determine whether NPTX2 can regulate EOC tumorigenesis *in vivo*, we injected EOC cell lines with NPTX2 overexpression, knockdown or control into the flank regions of nude mice, observed the tumor growths and followed the survival rates. At 5 weeks post-inoculation, the mean volume of the xenograft tumors in the NPTX2 overexpression group was obviously larger than that in the control group (**Figures 8A, B**), while the opposite results were obtained after NPTX2 knockdown (**Figures 8D, E**). In agreement, the mean weight of the tumors extracted from the NPTX2 overexpression group was obviously larger than the control group (**Figure 8C**), while the mean weight of the tumors extracted from the NPTX2 knockdown group was smaller than the control group (**Figure 8F**). Moreover, IHC was performed to detect the effects of NPTX2 overexpression or knockdown on tumor tissues. In the NPTX2 overexpression group, the staining intensity and expression levels of Ki-67 were all upregulated, while the opposite results were obtained in the NPTX2knockdown group (**Figure 8G**). A schematic diagram showing that NPTX2 overexpression promotes malignant phenotype of epithelial ovarian carcinoma *via* IL6-JAK2/STAT3 signaling pathway under hypoxia (**Figure 8H**). Taken together, these results suggested that NPTX2 regulates epithelial ovarian carcinoma tumorigenesis in nude mice.

## Discussion

In the present study, we found for the first time that NPTX2 was significantly upregulated in epithelial ovarian cancer and correlated with the poor prognosis. Since NPTX2 was the top regulated genes among TCGA and four GEO databases (GSE10971, GSE18520, GSE105437, and GSE26712), it is worth discussing whether NPTX2 play a vital role in EOC. We furtherly confirmed the obvious overexpression of NPTX2 in our clinical human EOC tissues and cultured cell lines.

In order to find the possible role of NPTX2 on EOC, we first searched the available literature about the roles of NPTX2 in malignant tumors and NPTX2 was reported mainly participated in five cancers, including glioma, pancreaticobiliary cancer, colorectal cancer, renal cell carcinoma and neuroblastoma (16–21). However, NPXT2 plays a contradictory role in those tumors. AlthoughNPTX2 showed a lower expression due to its promoter hypermethylation and identified poor prognosis in glioma and pancreaticobiliary cancer (12, 22), only one study demonstrated the direct effect of NPTX2 on the inhibiting proliferation and invasion of pancreatic cancer cells (11). As for colorectal cancer, renal cell carcinoma and neuroblastoma, all studies demonstrate the direct carcinogenesis effects of NPTX2. NPTX2 promotes colorectal cancer growth and liver metastasis by activating the canonical Wnt/β-catenin pathway *via* FZD6 (9). MiR-96 suppresses the proliferation, migration, and invasion of renal cell carcinoma *via* inhibiting NPTX2 (10). Targeting NPTX2 with the selected peptide can reduce the tumor burden in orthotopic mouse models of human neuroblastoma (20).

Therefore, we prefer to recognize NPTX2 as an possible oncogene in tumors. Our study also confirmed that NPTX2 overexpression could promote the proliferation, invasion and migration of EOC *in vitro* and *in vivo*, while NPTX2 knockdown can inhibit those malignant phenotypes of EOC.

To further explore the possible downstream mechanism of NPTX2 on the malignant phenotype of EOC, we performed GSEA on the TCGA dataset, and the results showed a significant IL6-JAK2/STAT3 signaling pathway enrichment in the higher NPTX2 expression group. IL6-JAK2/STAT3 signaling pathway is one of the most typical oncogenic signaling pathways in cancers. For example, NFAT1-regulated IL6 signaling contributes to the aggressive phenotypes of glioma (23). Cancer-associated fibroblasts promote gallbladder cancer growth *via* activation of the IL6-JAK/STAT3 signal pathway (24). Ovarian carcinoma-associated mesenchymal stem cells can activate the tumor cell stemness *via* activating IL6/STAT3 signaling (25). In our study, we demonstrated that NPTX2 overexpression could promote the expression and secretion of IL6, followed by JAK2/STAT3 signaling pathway activation in epithelial EOC. Moreover, after treatment withIL-6-neutralizing antibody, NPTX2 induced malignant phenotype of EOC was reversed. Therefore, these results demonstrate that NPTX2 promotes EOC’s malignant phenotype *via* upregulation of IL6 and the activation of the JAK2/STAT3 signaling pathway.

Due to the rapid growth of tumor cells, it is frequent to occur in ischemia and hypoxia (26). Hypoxia can promote the proliferation, invasion and migration of tumors through upregulation of HIF-1 (27). HIF-1 is the key transcription factor under hypoxia and is involved in the malignant phenotype of ovary cancers (28). For example, HIF-1 mediates epidermal growth factor-induced down-regulation of E-cadherin expression and cell invasion in human ovarian cancer cells (29). In our study, GSEA analysis indicated that NPTX2 might be correlated with a hypoxia environment. Then we inferred whether HIF1 could transcriptionally regulate the expression of NPTX2 under hypoxia. Both the luciferase reporter gene assays and ChIP assays demonstrated that HIF-1 directly transcriptionally regulates NPTX2 expression in EOC under hypoxic conditions. Furtherly, all the *in vitro* assays demonstrated that hypoxia can promote the proliferation, invasion and migration of EOC, while NPTX2 knockdown obviously abolished these promoting effects.

In conclusion, we found for the first time that NPTX2 was significantly upregulated in EOC tissues and correlated with the poor prognosis. NPTX2 overexpression can promote the proliferation and tumorigenesis of EOC *via* IL6-JAK2/STAT3 signaling pathway. Moreover, the expression of NPTX2 may be upregulated *via* HIF-1 due to the hypoxia environment caused by EOC’s rapid growth. Therefore, NPTX2 may be a promising treatment target for EOC molecular therapy.

## Data Availability Statement

Publicly available datasets were analyzed in this study. This data can be found here: The Cancer Genome Atlas (TCGA, http://cancergenome.nih.gov) and Gene Expression Omnibus (GEO, GSE10971, GSE18520, GSE105437 and GSE26712).

## Ethics Statement

The animal study was reviewed and approved by Human Ethics Committee of FUSCC.

## Author Contributions

XH participated in the design of the study. XH, XL, and LX performed the experiments and collected the data. YL performed the bioinformatics analysis and analyzed the data. HW and ZF produced the main draft of the text and the figures. XH and XJ contributed to data collection and the interpretation of data. XH wrote the manuscript. XC and XW conceived the project and supervised the research. All authors contributed to the article and approved the submitted version.

## Funding

This work was sponsored by grants from the Hospital Internal Foundation of Fudan University Shanghai Cancer Center (Grants. YJQN201927) and Eyas Program Foundation of Shanghai Anticancer Association (Grants. HYXH202021).

## Conflict of Interest

The authors declare that the research was conducted in the absence of any commercial or financial relationships that could be construed as a potential conflict of interest.
