# Supplementary material for: Overexpression of NPTX2 Promotes Malignant Phenotype of Epithelial Ovarian Carcinoma via IL6-JAK2/STAT3 Signaling Pathway Under Hypoxia
Source: Front Oncol. 2021 Mar 9;11:643986. doi: 10.3389/fonc.2021.643986 (PMC7985451; doi:10.3389/fonc.2021.643986)
Supplement: Supplementary file 1 [file Table_1.docx]

**Supplementary Table 1.**

**The clinical features of 45 cases of epithelial ovarian cancer patients.**

| **Clinical features** | | |
| --- | --- | --- |
| Age(median, range) | | 55(39-68) |
| Pathological classification | |  |
| high-grade serous carcinoma | | 45(100%) |
| FIGO stage | IB | 1(2.2%) |
|  | IIB | 6(13.3%) |
|  | IIIB | 13(28.9%) |
|  | IIIC | 25(55.6%) |
| CA-125 (0-35U/ml) (median, range) | | 846.3(89.5-＞5000) |
| HE4 (0-84.4pmol/L) (median, range) | | 398.7(51.2-＞1500) |
| CEA (0-5.2ng/ml) (median, range) | | 1.35(＜0.2-10.08) |
| CA19-9 (0-27U/ml) (median, range) | | 13.23(0.86-88.73) |
